# Combined machine learning identification and experimental validation of NDUFS8 and SUCLG1 as potential biomarkers for mechanical asphyxia

**DOI:** 10.3389/fmed.2026.1861399

**Published:** 2026-08-26

**Authors:** Hanfeng Jiang, Yi Shi, Xinbiao Liao, Dongchuan Zhang, Wencan Li, Lu Tian, Bi Xiao, Yu Shao, Tianpu Wu, Yue Chen, Weigang Zhang, Kaijun Ma, Hongmei Xu, Long Chen

**Affiliations:** 1 Department of Forensic Medicine, School of Basic Medical Sciences, Fudan University, Shanghai, China; 2 School of Forensic Medicine and Science, Fudan University, Shanghai, China; 3 Key Laboratory of Forensic Pathology, Guangdong Public Security Department, Guangzhou, China; 4 Institute of Criminal Science and Technology, Shanghai Public Security Bureau, Shanghai, China; 5 Institute of Criminal Science and Technology, Pudong Branch of Shanghai Municipal Public Security Bureau, Shanghai, China

**Keywords:** forensic pathology, machine learning, mechanical asphyxia, mitochondria, proteomics

## Abstract

**Introduction:**

Mechanical asphyxia (MA) is a major form of violent death caused by external forces that impair respiration and gas exchange. Despite its frequency in forensic casework, its diagnosis remains challenging because autopsy findings are often non-specific.

**Methods:**

In this exploratory study, we combined data-independent acquisition (DIA) proteomics with machine-learning methods to identify candidate myocardial markers of MA. A discovery cohort of human left ventricular myocardium was profiled to identify nominally differentially abundant proteins. The leading candidates were confirmed by Western blot and immunohistochemistry and further supported by animal models.

**Results:**

Two proteins central to mitochondrial energy metabolism, NDUFS8 and SUCLG1, emerged as candidate markers, and both were consistently downregulated in the MA group relative to controls.

**Discussion:**

These changes point to perturbed mitochondrial bioenergetics and oxidative-stress regulation as features of MA-associated myocardial injury. NDUFS8 and SUCLG1 may thus serve as candidate ancillary markers for mechanical asphyxia.

## Introduction

Death from mechanical asphyxia (MA) is a form of asphyxial death caused by external forces that obstruct respiration and impair gas exchange, encompassing hanging, ligature strangulation, manual strangulation, smothering, and choking (1, 2). MA accounts for a substantial proportion of violent deaths among women, making it an important concern in forensic practice (3, 4). Nevertheless, diagnosing MA remains challenging. Classical autopsy findings, including Tardieu spots, petechial hemorrhages, and the associated histopathological changes, are non-specific and frequently overlap with those of other causes of death, and in some cases overt anatomical signs are absent altogether (5, 6). This lack of specificity limits the accurate determination of the cause of death and highlights the need for objective molecular markers of MA.

Research on molecular markers of mechanical asphyxia has advanced considerably in recent years. Untargeted metabolomics, proteomics, Raman spectroscopy, and Fourier-transform infrared (FTIR) spectroscopy have each been used to characterize molecular alterations associated with MA and to distinguish it from other causes of death (7–14). Nevertheless, the forensic application of these findings remains constrained. In routine casework, samples are affected by the postmortem interval, environmental temperature, decomposition, and tissue autolysis. These factors can compromise molecular stability and analytical reproducibility. In addition, many studies rely on experimental models and lack adequate validation in human tissue. Among current omics strategies, proteomics is particularly suited to forensic biomarker discovery. It quantifies proteins directly and is less vulnerable to postmortem degradation than transcriptomics. It also yields more stable markers than metabolomics under postmortem conditions (15, 16). Data-independent acquisition (DIA) proteomics further combines broad proteome coverage with high reproducibility. This makes it well suited to the analysis of autopsy tissue and the identification of candidate markers of mechanical asphyxia (17–19).

Proteomic studies of mechanical asphyxia remain scarce. Several recent investigations have reported alterations in myocardial proteins associated with MA (12, 13), but systematic analyses in human forensic samples are still lacking, and the resulting high-dimensional data pose additional challenges for candidate selection. Random forest (RF) and the least absolute shrinkage and selection operator (LASSO) are widely used for dimensionality reduction and feature selection (20–22). RF is an ensemble method that captures nonlinear and higher-order relationships in high-dimensional data (23). It also provides variable-importance estimates and is relatively robust to overfitting (24). LASSO is a penalized regression method that performs sparse feature selection by shrinking coefficients toward zero (25). Combining the two can improve the parsimony, stability, and interpretability of the selected feature set (26).

Mitochondria are a key target in mechanical asphyxia. Prolonged hypoxia impairs mitochondrial energy metabolism, dissipates the membrane potential, and disturbs reactive oxygen species homeostasis. These changes are closely linked to the pathophysiology of asphyxia. Previous studies, including our own, have reported altered expression of several mitochondria-related proteins in MA, including hypoxia-inducible factor-1α (HIF-1α), 8-hydroxyguanine DNA glycosylase (OGG1), C/EBP homologous protein (CHOP), and hydroxyacyl-CoA dehydrogenase (HADH) (27–31). Together, these findings support integrating mitochondria-focused proteomics with machine learning to identify candidate markers of MA.

In this study, we performed data-independent acquisition (DIA) proteomic analysis of left ventricular myocardium from human cases of mechanical asphyxia. Integrated bioinformatic and machine-learning analyses identified two candidate mitochondria-related proteins. These were the alpha subunit of succinate–CoA ligase (SUCLG1) and NADH:ubiquinone oxidoreductase core subunit S8 (NDUFS8). Both were further examined in human forensic samples. Their downregulation was broadly consistent with previous observations and extended these to the proteome level. Overall, our findings link dysregulation of mitochondrial proteins to mechanical asphyxia and suggest that SUCLG1 and NDUFS8 may serve as candidate ancillary markers of MA.

## Methods and materials

### Animal sample

In this investigation, male C57BL/6J mice (weighing 20 ± 5 g) were employed. All animal-related experimental techniques were approved by the Fudan University Animal Ethics Committee and conducted in accordance with institutional guidelines. Sodium pentobarbital (45 mg/kg) was administered intraperitoneally to anesthetize the mice. After adequate anesthesia, the animals were maintained at room temperature (25 °C). Three postmortem-interval (PMI) groups (0, 6, and 12 h) and six cause-of-death groups were studied, each comprising five mice (*n* = 90). The control groups comprised drowning, hemorrhagic shock, brain injury, and carbon monoxide poisoning: drowning was induced by immersion in water until death; hemorrhagic shock by transection of the common carotid artery; brain injury with a free-fall weight device; and carbon monoxide poisoning by housing mice in a sealed chamber with continuous carbon monoxide delivery at 14 ml/min for 120 min. The experimental groups comprised hanging and manual strangulation. For hanging, a thin cord was looped around the neck under anesthesia and the animal was suspended for 5 min, with a 100-g weight attached to the tail; for manual strangulation, sustained external compression of the larynx was applied for 5 min. Continuous electrocardiographic monitoring was performed in all groups using a BL-420 system (Taimeng), and death was confirmed by the cessation of cardiac electrical activity. To assess whether sodium pentobarbital anesthesia affects the expression of mitochondria-related proteins in the myocardium, mice were randomly assigned to a control (CON) group and a pentobarbital (PB) group (*n* = 6 per group). CON mice were euthanised directly, whereas PB mice were euthanised after 5 min of intraperitoneal sodium pentobarbital anesthesia (45 mg/kg). Left ventricular myocardium was then collected.

### Human sample

Human myocardial specimens were collected at the Forensic Science Center of Fudan University, Shanghai. In total, 52 Western blot specimens were obtained between 2014 and 2025 and 30 immunohistochemistry specimens between 2022 and 2026; the two cohorts were independent and did not overlap. The 82 specimens comprised 31 mechanical asphyxia cases and 51 controls. Data-independent acquisition (DIA) proteomic analysis was performed on 12 specimens drawn from the Western blot cohort, so that the Western blot analysis served as an orthogonal confirmation of the proteomic findings within the same cohort. All specimens were stored at −80 °C, and tissues showing autolysis or degradation were excluded. Mechanical asphyxia was diagnosed from the forensic autopsy, characteristic asphyxial features, toxicological analysis, death-scene investigation and case history, together with exclusion of all other possible causes of death. Each diagnosis was made independently by three forensic pathologists using an exclusion-based approach. Specimen collection was approved by the Ethics Review Committee of the School of Basic Medical Sciences, Fudan University, and conducted in accordance with the Declaration of Helsinki. The characteristics of all 82 specimens are summarized in Table 1, with per-case information provided in Supplementary Data 1.

**Table 1:** Summary table of human sample information.

Category	MA (*n* = 31)	HS (*n* = 19)	BI (*n* = 20)	OC (*n* = 12)
Age, years	39.7 ± 17.4	46.9 ± 19.7	52.7 ± 17.3	34.5 ± 21.1
Gender, male (%)	7 (22.6 %)	12 (63.2%)	13 (65.0%)	7 (58.3%)
PMI, hour	12.6 ± 8.2	3.0 ± 3.6	6.0 ± 4.2	7.9 ± 12.9
Presence of fatal COHb %	0 (0.0%)	0 (0.0%)	0 (0.0%)	2 (16.7%)
Blood alcohol (>0.2 mg/ml)	2 (6.3%)	2 (10.5%)	0 (0.0%)	0 (0.0%)
Other toxic substances detected	0 (0.0%)	0 (0.0%)	0 (0.0%)	0 (0.0%)

MA, mechanical asphyxia; HS, hemorrhagic shock; BI, brain injury; OC, other causes of death; PMI, postmortem interval.

### Proteomics

#### Protein extraction and trypsin digestion

Phosphate-buffered saline (PBS) was used three times to wash tissue samples in order to remove any remaining blood or debris. Following cleaning, the samples were sliced into tiny pieces and lysed in 8.1 M urea/100 mM Tris–HCl buffer (pH 8.0) with phosphatase and protease inhibitors added. Using cycles of 3 s on and 3 s off at 25% amplitude, the lysates were homogenized by sonication for 1 min. The supernatants were obtained as total extracts following a 10-min centrifugation at 11,500 × g. The Bradford technique was used to determine the protein content.

210 μg of protein from each sample was reduced with 10 mM dithiothreitol (DTT) at 57 °C for 35 min before being alkylated with 10 mM iodo-acetamide (IAA) at room temperature for 35 min in the dark for enzymatic digestion. After processing, the protein samples were put onto centrifugal filters and combined with 200 μL of 8 M urea made with 100 mM Tris–HCl. The samples were gently vortexed and then centrifuged for 25 min at 11,500 × g. To get rid of any remaining urea and impurities, the filter membranes were cleaned three times with 210 μL of UA buffer and then three more times with 200 μL of 50 mM ammonium bicarbonate (NH4HCO3). Using 4 μg of sequencing-grade trypsin (Promega) in 100 μL of 50 mM NH4HCO3, proteins that remained on the filters were broken down overnight at 37.5 °C. Centrifugation was used to gather the resultant peptides, which were subsequently fully dried in a vacuum concentrator (Concentrator Plus, Eppendorf).

#### Nano-LC–MS/MS analysis

Peptide samples were subjected to proteome profiling using an EASY nLC1200 ultra-high-performance liquid chromatography system (Thermo Fisher Scientific) interfaced with an OE480 Hybrid Quadrupole-Orbitrap Mass Spectrometer (Thermo Fisher Scientific). Dried peptides were initially placed onto a self-packed trap column (2 cm × 100 μm inner diameter) containing 3 μm ReproSil-Pur C18-AQ beads (Dr. Maisch GmbH) after being reconstituted in Solvent A (0.1% formic acid in water). A self-packed analytical column (10 cm × 75 μm inner diameter) filled with 1.9 μm ReproSil-Pur C18-AQ beads (Dr. Maisch GmbH) was then used to perform peptide separation. Before being added to the mass spectrometer, the eluted peptides were ionized at 2.1 kV. Data-independent acquisition (DIA) mode was used for mass spectrometric acquisition. In particular, full MS scans were acquired over an m/z range of 300–1,400 at a resolution of 60,000. This was followed by 30 DIA windows collected at a resolution of 15,000. Fragmentation was performed by higher-energy collisional dissociation (HCD) with the normalized collision energy set to 30%. The default charge state for MS2 was set to 3, and all spectra were obtained in profile mode.

### Data analysis

The iProteome one-stop cloud platform was used to evaluate raw mass spectrometry data for both quantitative profiling and qualitative identification. The UniProt Homo sapiens proteome dataset (Taxon ID: 9606) was used for database retrieval. With a maximum of two missed cleavages, trypsin was chosen as the digestive enzyme. Acetylation at the protein's N-terminus and methionine oxidation were considered variable modifications, but carbamidomethylation on cysteine was considered a fixed modification. The mass tolerances for precursors and fragments were set at 20 ppm and 0.05 Da, respectively. Protein abundance was normalized within each sample using the Fraction of Total (FOT) method, in which each protein's signal was expressed as a proportion of the total quantitative signal within that sample and scaled by 1 × 105 (iFOT). Data quality was assessed before downstream analysis. The number of peptides identified per protein was summarized to evaluate identification reliability, and the per-sample distribution of log10-transformed FOT values was visualized as box plots to assess quantitative consistency across the 12 discovery samples (Supplementary Figure S1). Missing FOT values were imputed with the minimum FOT value across all detected proteins. To limit the influence of imputation, only proteins with no more than one imputed value per group were retained. FOT values were then log2-transformed for all subsequent analyses.

### Bioinformatics analysis

Sample distribution and overall proteomic expression patterns were assessed by principal component analysis (PCA) in R after data normalization and mean centering. Nominally differentially abundant proteins were identified using Student's *t*-test. Nominally differentially abundant proteins identified from comparisons of mechanical asphyxia with hemorrhagic shock and with brain injury were intersected. Volcano plots and heatmaps were generated using the R packages “ggplot2” and “pheatmap”, respectively. Gene Ontology (GO) and KEGG pathway enrichment analyses of the intersected candidate proteins were performed using the R package “clusterProfiler”. Enrichment *P* values were adjusted for multiple testing using the Benjamini–Hochberg procedure, and terms with a q value < 0.05 were considered significantly enriched.

### Machine learning

Random forest analysis was performed with the randomForest package (ntree = 500, mtry = 3, variable importance ranked by MeanDecreaseGini). LASSO and elastic-net regression were performed with the glmnet package (family = “binomial”); α was fixed at 1 for LASSO, whereas for elastic net α was tuned from 0 to 1 by cross-validation and set to the optimal value of 0.4, with λ selected at λ1SE by five-fold cross-validation in all cases. All models were applied to the 12 mitochondria-related nominally differentially abundant proteins across the 12 samples, using log2-transformed abundances. Candidate biomarkers of mechanical asphyxia were defined by intersecting the maximal clique centrality (MCC) hub proteins with the proteins prioritized by random forest, LASSO, and elastic net. These candidates were then examined by single-protein gene set enrichment analysis (GSEA) to explore their potential biological roles.

### Western blot

Each sample's 30 mg of tissue was lysed in 500 μL of Radio-Immunoprecipitation Assay buffer (P0013C, Beyotime, China). For denaturation, proteins were combined with 5 × SDS loading buffer (P0015, Beyotime, China) and heated to 90 °C for 15 min. SDS-PAGE was used to separate samples at a concentration of 2 μg/μL before they were put onto methanol-activated PVDF membranes (IPVH00010, Merck, Germany). After blocking the membranes with 5% bovine serum albumin (BSA; ST023, Beyotime, China) in TBST for 1 h at room temperature, primary antibodies against SUCLG1 (86072-1-RR, Proteintech), NDUFS8 (25172-1-AP, Proteintech), and β-Actin (AF7018, Affinity) were incubated overnight at 4 °C. After five washes with TBST for 12 min each, the membranes were incubated with HRP-labeled secondary antibodies (1:5000) at 37 °C for 1.5 h. This was followed by additional TBST washes. Membranes were blocked once again and re-incubated with the appropriate secondary antibody after being subjected to stripping buffer (ZB382859, Thermo Scientific) for 15 min in order to identify NDUFS8 and SUCLG1. An enhanced chemiluminescence (ECL) substrate (RM00021P, ABclonal, China) was used to visualize the bands, and a Tanon 4600 chemiluminescence imaging equipment (Tanon, China) was used to record them.

### Immunohistochemistry

Tissues immersed in paraffin were cut into 3.5-μm slices, put on glass slides, and incubated for 2 h at 60 °C. After deparaffinization in xylene for three 10- min washes, the sections were rehydrated through a graded ethanol series of 100%, 95%, 85%, and 75%, with 5 min at each step. This was followed by three 5-min washes in PBST. Antigen retrieval was carried out by microwave heating at high power for 10 min in alkaline buffer (pH 9.0). Following cooling to 37 °C, the sections underwent three 5- min PBS washes. After quenching endogenous peroxidase activity for 30 min at 37 °C with 3% hydrogen peroxide, PBS rinses were performed. The sections were blocked with 5% BSA for 1 h at room temperature (37 °C) to lessen nonspecific staining. After applying primary antibodies against SUCLG1 (86072-1-RR, Proteintech) and NDUFS8 (25172-1-AP, Proteintech), the slides were incubated at 4 °C for the entire night. Following three PBS washes, the sections were incubated for 1 h at 37 °C with HRP-conjugated secondary antibodies (1:200) (S0001, Affinity). After more PBS washes, DAB was used to induce color development. Once the proper staining was seen under a light microscope, the reaction was stopped using tap water. Hematoxylin was used as a counterstain for 3 min, followed by 3 sec of differentiation in 1% acid alcohol and a water rinse. The slides were then covered with neutral resin and coverslips after being dehydrated using graded ethanol and cleaned in xylene three times for 10 min each. Both SUCLG1 and NDUFS8 immunoreactivity were localized predominantly to the cytoplasm of cardiomyocytes. Immunohistochemical images were acquired using a HELA60 microscope imaging system (Zoeheal, Shanghai), and all slides were digitized at 40 × magnification. For each section, six random fields were selected at 200× magnification and analyzed using ImageJ version 1.54 g (National Institutes of Health, Bethesda, MD, USA). Images were converted to 8-bit and calibrated to optical density. A batch macro then applied the same calibration and intensity threshold to all images. This defined the DAB-positive area after non-cardiac tissue and blank regions had been excluded. Staining intensity was expressed as the average optical density (AOD), defined as the integrated optical density divided by the corresponding tissue area and averaged across the six fields. The mean of the six fields was taken as the immunohistochemical staining intensity of that sample. All quantification was performed blinded to group allocation. The individual case was used as the statistical unit rather than the individual field. Because quantification was fully automated using preset parameters, which excludes subjective scoring, inter-observer agreement was not assessed. For negative controls, the primary antibody was replaced with PBS while all other steps were kept identical, and no specific staining was observed (Supplementary Figure S4).

### Statistical analysis

GraphPad Prism 9.0 (GraphPad Software, La Jolla, CA, USA) and IBM SPSS Statistics version 27 (IBM Corp., Armonk, NY, USA) were used for statistical analysis. Data are presented as the mean ± standard deviation (SD). Comparisons between two groups were performed using an unpaired Student's *t*-test. Comparisons among multiple groups were performed using one-way ANOVA followed by Tukey's multiple-comparisons test. The discriminatory performance of NDUFS8 and SUCLG1 was evaluated using receiver operating characteristic (ROC) curve analysis, and the combined score was generated using binary logistic regression in IBM SPSS Statistics version 27. A two-sided *p* value < 0.05 was considered statistically significant.

## Results

### Exploratory identification of nominally differentially abundant proteins between MA and control groups

**Figure 1:**
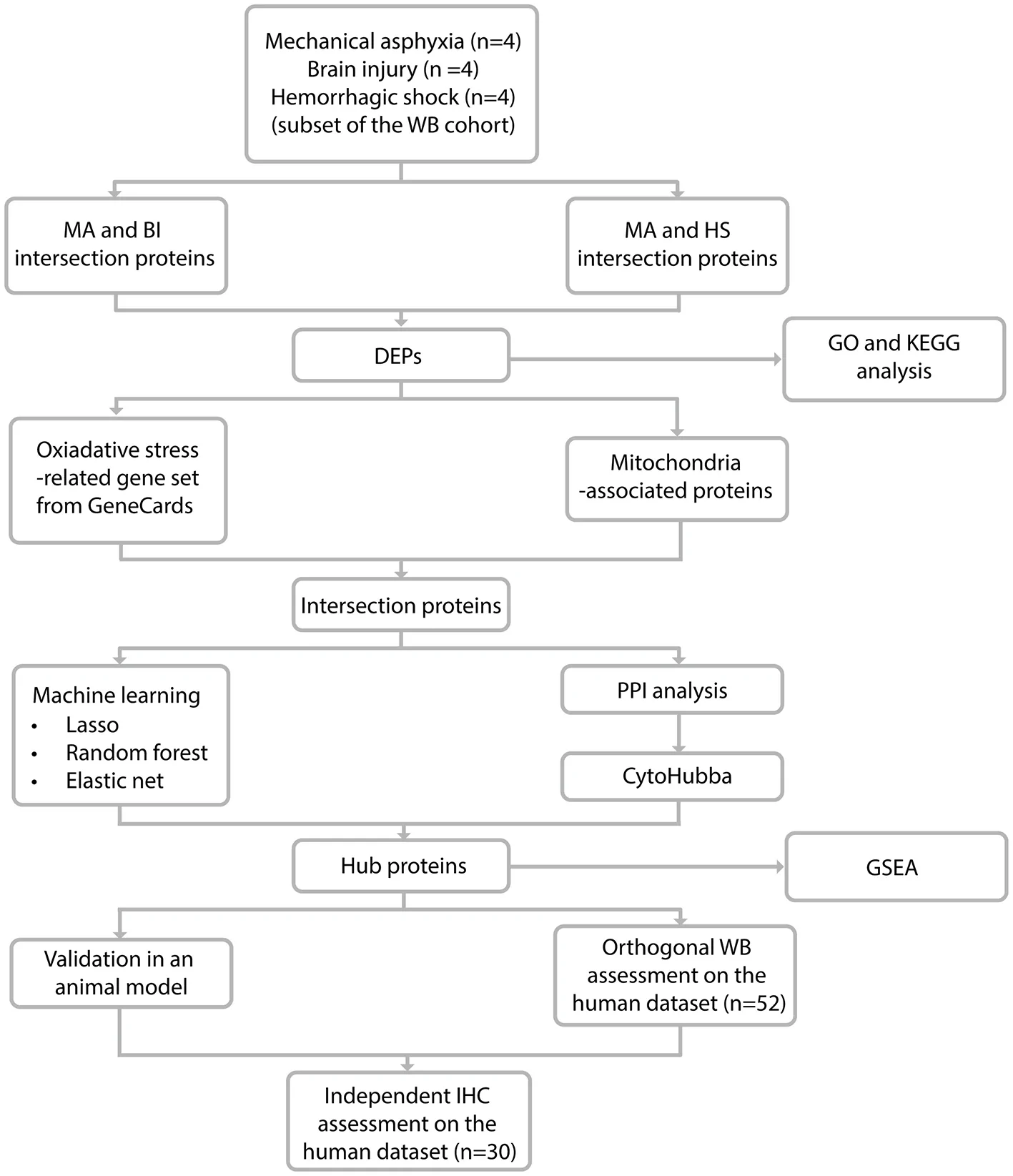
Flowchart of the bioinformatic analysis of candidate proteins. The study included 82 human samples: a 52-case Western blot cohort and an independent, non-overlapping 30-case immunohistochemistry cohort. The 12 samples used for discovery proteomics were drawn from the 52-case Western blot cohort. Western blotting therefore provided an orthogonal assessment in the larger overlapping cohort, whereas immunohistochemistry provided an independent assessment in a non-overlapping cohort.

**Figure 2:**
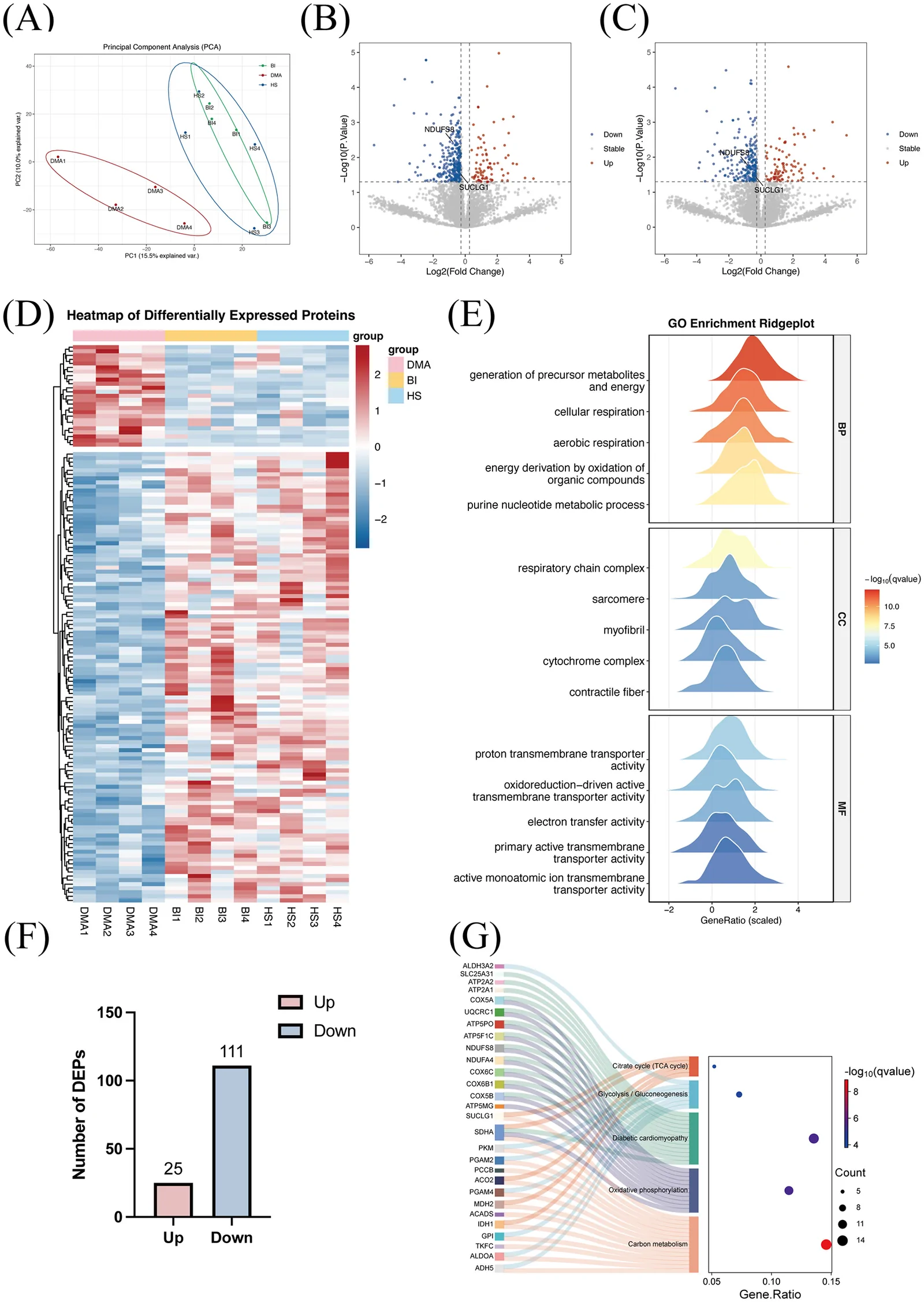
Preliminary identification of nominally differentially abundant proteins in the proteomic analysis of human myocardial tissue. **(A)** Principal component analysis (PCA) of 4,224 quantified proteins. **(B)** Nominally differentially abundant proteins between the MA and BI groups. **(C)** Nominally differentially abundant proteins between the MA and HS groups. **(D)** Heatmap of the nominally differentially abundant proteins. **(E)** Ridge plot of the GO enrichment analysis of the 136 nominally differentially abundant proteins. **(F)** Bar plot showing 25 upregulated and 111 downregulated proteins among the nominally differentially abundant proteins. **(G)** Sankey diagram of the KEGG pathway enrichment analysis of the 136 proteins. The KEGG enrichment plot was generated using https://www.bioinformatics.com.cn, an online platform for data analysis and visualization.

Figure 1 outlines the bioinformatic workflow of this study. DIA proteomic analysis was performed on left ventricular myocardium from four cases in each of the mechanical asphyxia (MA), hemorrhagic shock (HS), and brain injury (BI) groups. A total of 9,870 proteins were identified, of which 4,224 were retained for analysis after filtering based on missingness. Principal component analysis showed clear separation of the MA group from both control groups (Figure 2A). In this exploratory analysis, proteins with a fold change > 1.2 or < 1/1.2 and nominal *p* < 0.05 were considered nominally differentially abundant proteins for further investigation. Because of the small, high-dimensional discovery dataset, no candidate remained significant after FDR correction, and nominal *P* values were therefore used only for exploratory screening. Nominal Student's *t*-test *P* values were therefore used for preliminary candidate screening, followed by orthogonal experimental assessment. The MA vs. BI comparison yielded 84 upregulated and 297 downregulated proteins (Figure 2B). The MA vs. HS comparison yielded 90 upregulated and 229 downregulated proteins (Figure 2C). Hierarchical clustering further showed distinct expression patterns across the three groups (Figure 2D). Intersection of the two sets of nominally differentially abundant proteins gave 25 upregulated and 111 downregulated proteins (Figure 2F).

These 136 nominally differentially abundant proteins were then subjected to GO and KEGG enrichment analyses. Detailed information on these proteins is provided in Supplementary Data 3. GO analysis showed that the generation of precursor metabolites and energy, together with cellular respiration, were the most enriched biological processes. The enriched cellular components centered on the respiratory chain complex, cytochrome complex, and sarcomere, and the main molecular functions were proton transmembrane transporter activity and electron transfer activity (Figure 2E). KEGG analysis indicated that these proteins were mainly involved in the citrate cycle, glycolysis/gluconeogenesis, oxidative phosphorylation, and carbon metabolism (Figure 2G).

### Prioritization of candidate proteins through bioinformatic and machine-learning analyses

**Figure 3:**
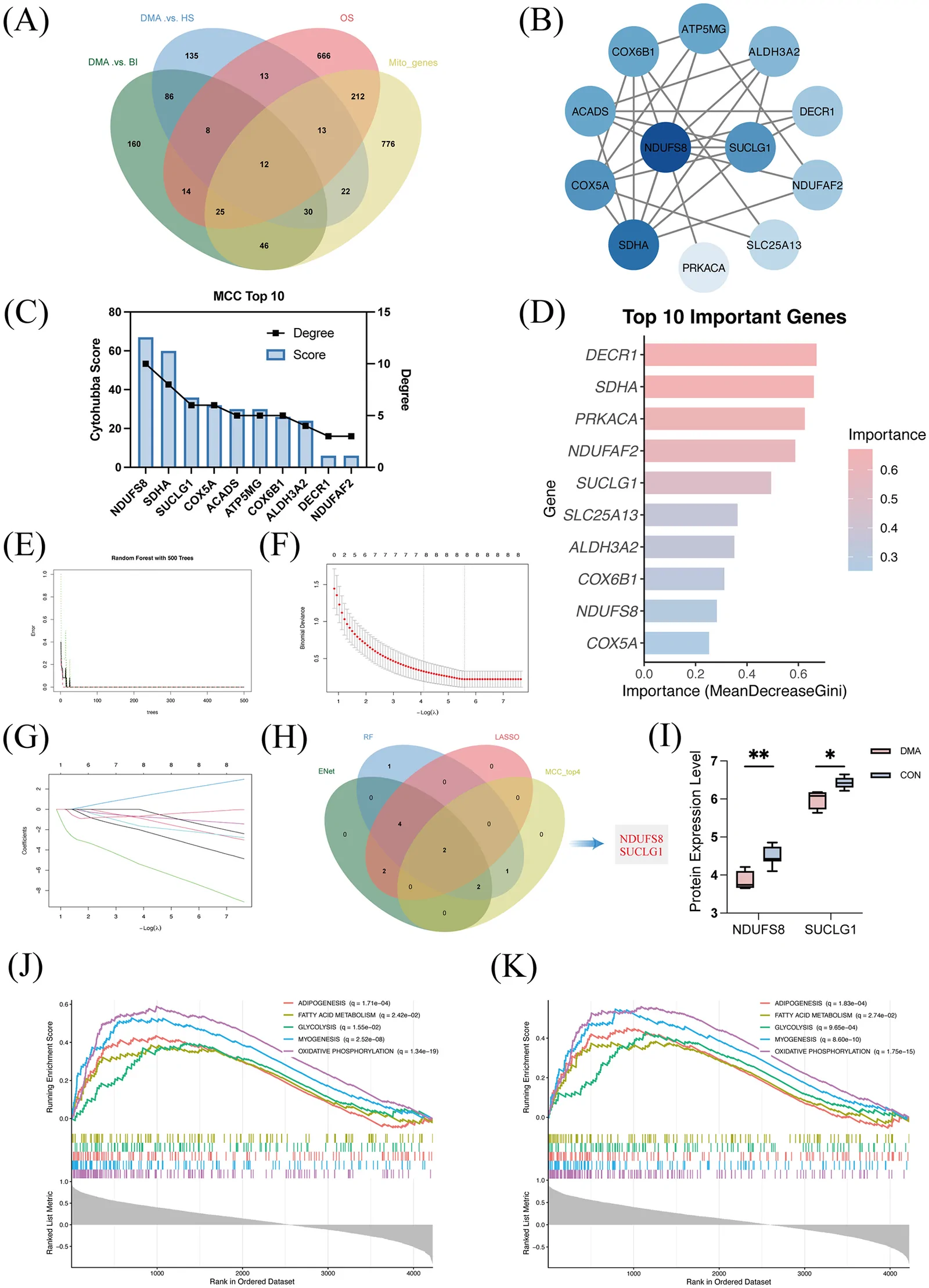
Identification of SUCLG1 and NDUFS8 as potential biomarkers of mechanical asphyxia. **(A)** Intersection analysis of the nominally differentially abundant proteins, oxidative stress–related genes, and mitochondria-related proteins. **(B)** Protein–protein interaction (PPI) network constructed from the 12 mitochondria- and oxidative stress–related proteins. **(C)** The top 10 hub proteins identified by the cytoHubba plugin. The node degree is shown as the line plot, and the cytoHubba scores from the maximal clique centrality (MCC) algorithm are shown as the bar chart. **(D)** Ranking of variable importance in the random forest model based on the mean decrease in the Gini index. **(E)** Error rates of the random forest model as a function of the number of trees. **(F)** Selection of the optimal penalty parameter (λ) in the LASSO model by cross-validation, with binomial deviance plotted against –log(λ). **(G)** LASSO coefficient profiles of the candidate proteins plotted against –log(λ). **(H)** Venn diagram showing the intersection among the three machine-learning methods (random forest, LASSO, and elastic net) and the top MCC-ranked hub proteins, which identified the core candidate proteins. **(I)** Expression levels of NDUFS8 and SUCLG1 in the proteomic dataset, analyzed by Student's *t*-test. MA, mechanical asphyxia; CON, control group; *p < 0.05, **p < 0.01. **(J)** Single-protein gene set enrichment analysis (GSEA) of NDUFS8. **(K)** Single-protein GSEA of SUCLG1.

Mechanical asphyxia is an acute hypoxic process closely linked to oxidative stress and to mitochondria, the principal organelles of cellular respiration. The 136 nominally differentially abundant proteins were cross-referenced with 963 oxidative stress–related genes from GeneCards and with mitochondria-associated proteins from MitoCarta3.0, yielding 12 mitochondria- and oxidative stress–related proteins (Figure 3A). These proteins were mapped in STRING to construct a protein–protein interaction network comprising 12 nodes and 29 edges (Figure 3B). Maximal clique centrality (MCC) in cytoHubba ranked the ten hub proteins as NDUFS8, SDHA, SUCLG1, COX5A, ACADS, ATP5MG, COX6B1, ALDH3A2, DECR1, and NDUFAF2 (Figure 3C). As exploratory feature-prioritization tools, random forest and elastic-net regression each ranked the top ten proteins by importance, whereas LASSO regression retained eight proteins with non-zero coefficients (Figures 3D–G; the elastic-net coefficient profiles and cross-validation curve are shown in Supplementary Figure S2). Intersecting the proteins prioritized by random forest, elastic net, and LASSO with the top MCC-ranked hub proteins identified NDUFS8 as the leading candidate. To avoid reliance on a single marker, we additionally retained SUCLG1, a highly ranked MCC hub protein that was likewise prioritized by all three machine-learning methods, giving two candidate markers, NDUFS8 and SUCLG1 (Figure 3H). Both were nominally downregulated in the mechanical asphyxia group relative to the control groups (Figure 3I). Single-protein GSEA showed that NDUFS8 and SUCLG1 were most strongly and positively enriched in oxidative phosphorylation (q = 1.34 × 10^−19^ and 1.75 × 10^−15^, respectively), together with myogenesis, glycolysis, adipogenesis, and fatty acid metabolism (Figures 3J, K).

### Assessment of candidate potential biomarkers in animal models at different postmortem intervals

**Figure 4:**
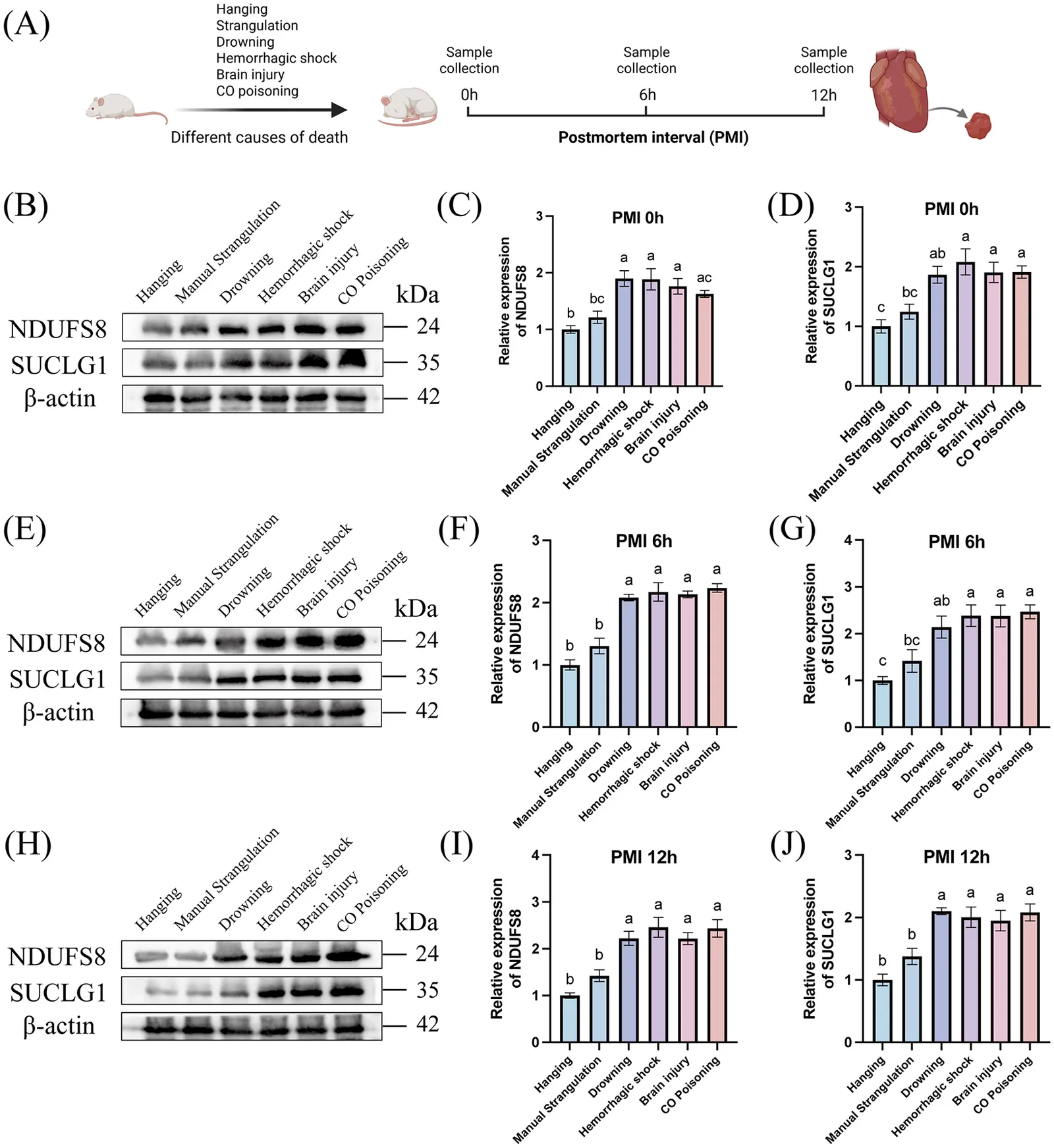
SUCLG1 and NDUFS8 were downregulated in the hanging and manual strangulation groups in the animal model. **(A)** Myocardial tissues were collected from mice subjected to six causes of death at three postmortem intervals (PMIs: 0, 6, and 12 h). **(B–D)** NDUFS8 and SUCLG1 expression at PMI 0 h was examined by western blotting and quantitative analysis. **(E–G)** The corresponding analyses were performed at PMI 6 h. **(H–J)** The corresponding analyses were performed at PMI 12 h. Causes of death were compared separately within each PMI using one-way ANOVA followed by Tukey's multiple-comparisons test. Significant differences are indicated by different lowercase letters (*p* < 0.05). Data are presented as mean ± SD; *n* = 5 mice per cause-of-death group at each PMI. Created in BioRender. Jiang, H. (2026) https://BioRender.com/jh68lrk.

The selected candidates were next validated in a mouse model of asphyxia. Myocardium was collected at postmortem intervals (PMIs) of 0, 6, and 12 h from mice subjected to six causes of death: hanging, manual strangulation, drowning, hemorrhagic shock, brain injury, and carbon monoxide poisoning. Protein levels were assessed by western blot (Figure 4A). At a PMI of 0 h, NDUFS8 was significantly lower in the hanging and manual strangulation groups than in the controls, although the manual strangulation and carbon monoxide poisoning groups did not differ. SUCLG1 was similarly reduced in the hanging and manual strangulation groups, with no significant difference between the manual strangulation and drowning groups (Figures 4B–D). At 6 h, NDUFS8 remained lower in the asphyxia groups than in the controls. SUCLG1 was also reduced in the asphyxia groups, although the drowning and manual strangulation groups did not differ significantly (Figures 4E–G). At 12 h, both NDUFS8 and SUCLG1 were markedly lower in the asphyxia groups than in the corresponding controls (Figures 4H–J). Thus, although some between-group differences were not significant at early PMIs, the downregulation of NDUFS8 and SUCLG1 in the asphyxia groups became more pronounced with increasing PMI. Sodium pentobarbital did not significantly alter the myocardial expression of Drp1, SUCLG1, VDAC, NDUFS8, or Tomm20 compared with the control group, suggesting that anesthesia had no appreciable effect on these markers under the present conditions (Supplementary Figure S3).

### Assessment of NDUFS8 and SUCLG1 as preliminary potential markers in human samples

**Figure 5:**
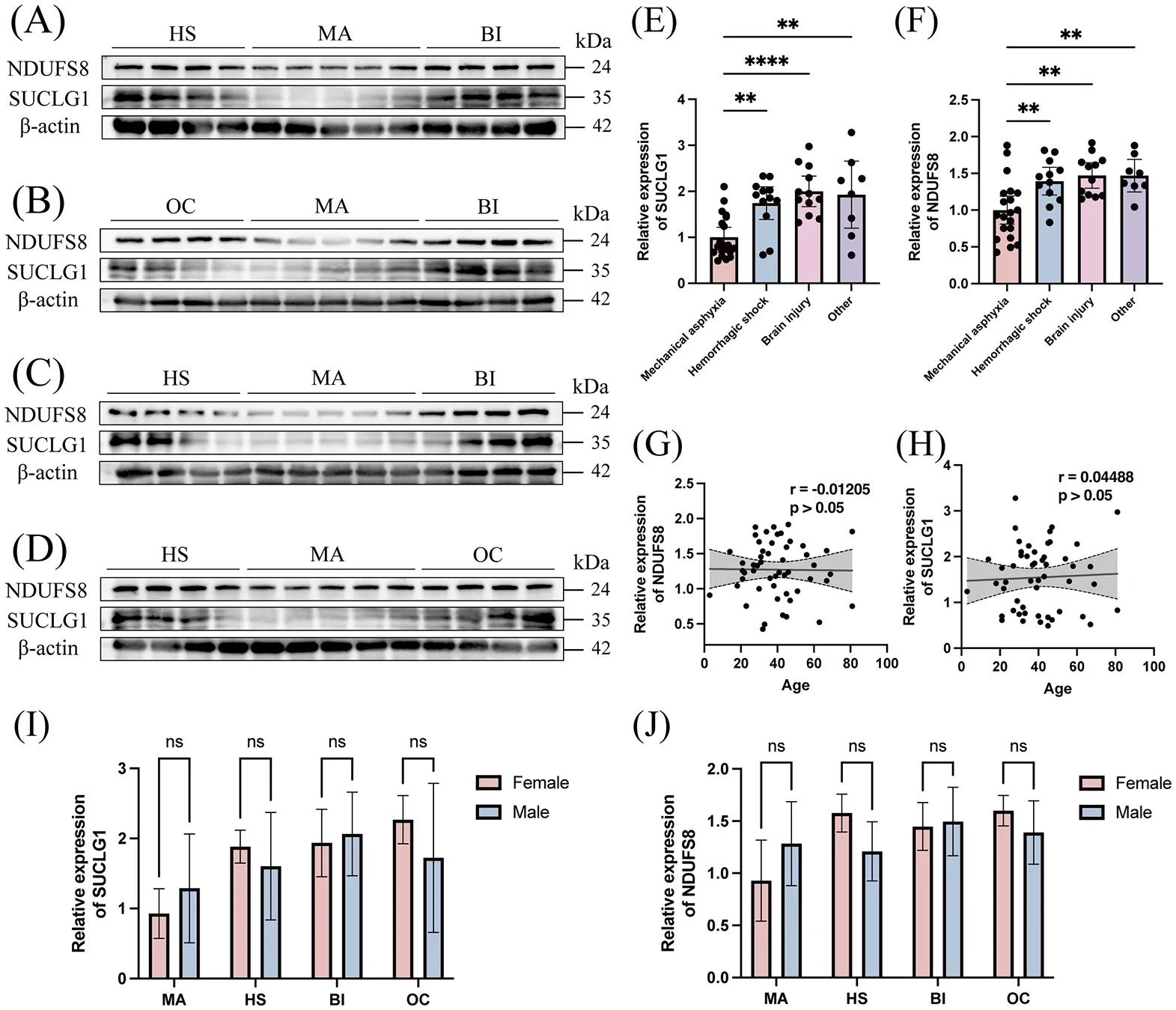
SUCLG1 and NDUFS8 were markedly downregulated in human samples from the mechanical asphyxia group. **(A–F)** Western blotting and quantitative analysis were performed to determine the expression levels of NDUFS8 and SUCLG1 in human myocardial tissues from different causes-of-death groups. **(G, H)** Correlation analyses were used to evaluate the associations of NDUFS8 and SUCLG1 expression with age. **(I)** The relationship between SUCLG1 expression and sex was analyzed within each group. **(J)** The association between NDUFS8 expression and sex was likewise examined in each group. Panels **(E, F)** were analyzed by one-way ANOVA followed by Tukey's multiple-comparisons test, whereas panels **(I, J)** were analyzed by two-way ANOVA followed by Tukey's multiple-comparisons test. Data are presented as mean ± SD. *p < 0.05, **p < 0.01, ***p < 0.001, ****p < 0.0001; ns, not significant. The solid line indicates the fitted linear regression line, and the surrounding shaded region represents the 95% confidence interval (CI). *P* values for Pearson's correlation coefficients (r) were calculated using a two-sided *t*-test. Analyses were based on 52 human myocardial samples, comprising 20 mechanical asphyxia, 12 hemorrhagic shock, 12 brain injury, and 8 other causes of death.

The expression patterns of NDUFS8 and SUCLG1 were further evaluated by western blotting in 52 human samples. The cohort comprised 20 mechanical asphyxia cases, 12 hemorrhagic shock cases, 12 brain injury cases, and 8 cases from other causes of death. Both NDUFS8 and SUCLG1 were markedly reduced in the mechanical asphyxia group relative to the controls (Figures 5A–F). We also assessed the influence of age and sex. Regression analysis showed no significant association between protein expression and age (Figures 5G, H), and no significant sex-related differences were found in any group (Figures 5I, J).

### Exploratory evaluation of the discriminatory ability of candidate markers for mechanical asphyxia in human samples

**Figure 6:**
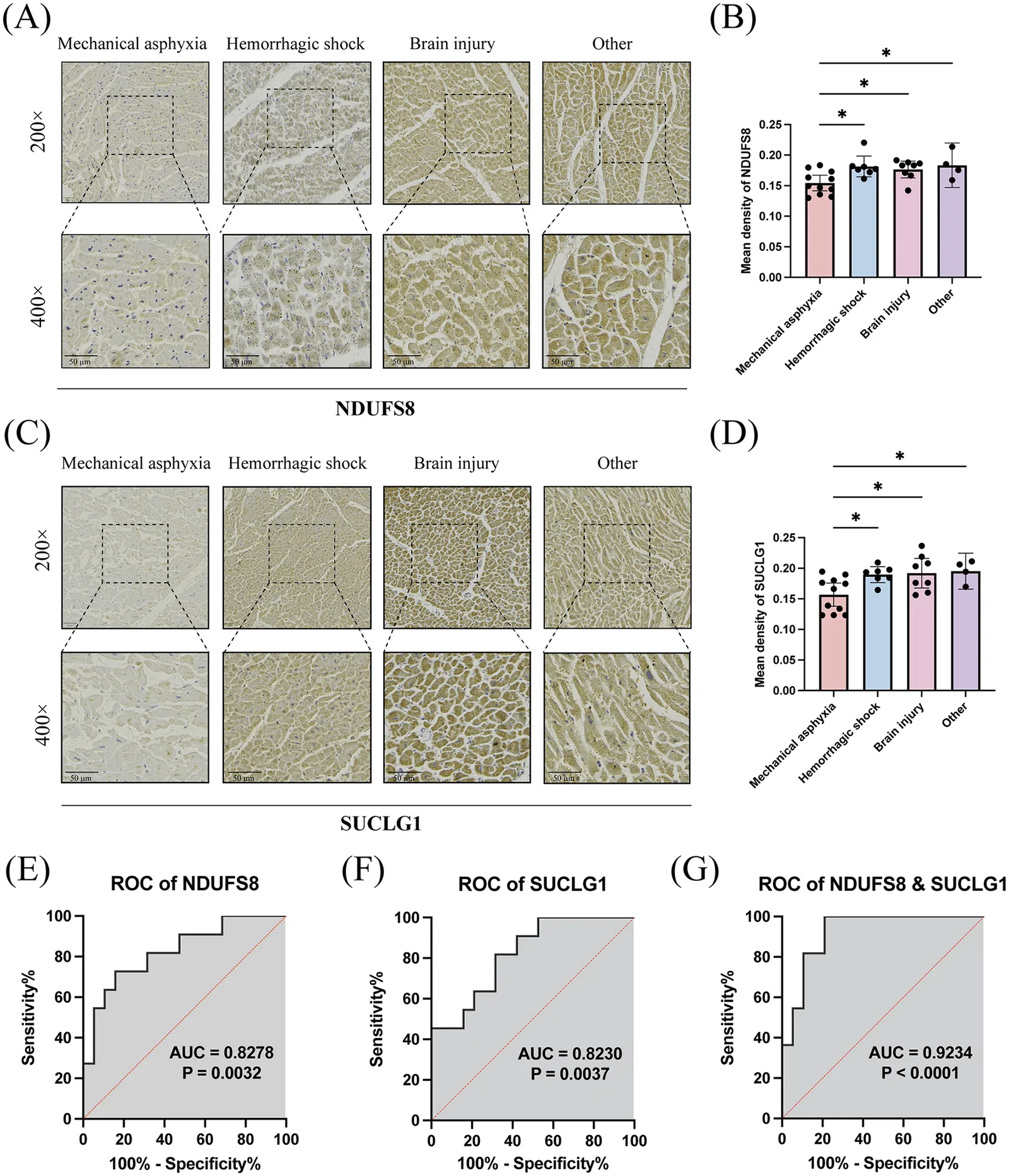
Assessment of NDUFS8 and SUCLG1 expression in human samples. **(A, B)** Representative immunohistochemical staining images of NDUFS8 and quantitative analysis of NDUFS8 staining across groups with different causes of death. **(C, D)** Representative immunohistochemical staining images of SUCLG1 and quantitative analysis of SUCLG1 staining across the same groups. NDUFS8 and SUCLG1 immunoreactivity was localized predominantly to the cytoplasm of cardiomyocytes. Staining intensity was quantified as the average optical density (AOD, integrated optical density divided by tissue area) from six random 200× fields per section using ImageJ with preset threshold parameters, blinded to group allocation, and the mean of the six fields was taken as the value for each case. Scale bars represent 200 μm. Negative controls in which the primary antibody was replaced with PBS showed no specific staining (Supplementary Figure S4). **(E)** ROC curve for NDUFS8. **(F)** ROC curve for SUCLG1. **(G)** ROC curve for the combined NDUFS8 and SUCLG1 model, in which the combined score was the predicted probability from a binary logistic regression of the two proteins fitted in IBM SPSS Statistics version 27. Differences in panels **(B)** and **(D)** were assessed by one-way ANOVA followed by Tukey's multiple-comparisons test (*p* < 0.05). Data are presented as mean ± SD, with the individual case as the statistical unit. Analyses were based on 30 human myocardial samples, comprising 11 mechanical asphyxia, 7 hemorrhagic shock, 8 brain injury, and 4 other causes of death.

We collected 30 human myocardial tissue samples and performed immunohistochemical staining to further assess the expression of NDUFS8 and SUCLG1. Consistent with the western blot results in both human and animal specimens, NDUFS8 and SUCLG1 expression was significantly lower in the mechanical asphyxia group than in the control groups (Figures 6A–D). Negative-control sections in which the primary antibody was replaced with PBS showed no specific staining, confirming the specificity of the immunohistochemical signal (Supplementary Figure S4). Receiver operating characteristic (ROC) analysis was then performed for NDUFS8 and SUCLG1 using the mean optical density values from the 30 samples. Due to the absence of significant differences in the expression of either biomarker among the individual control groups, cases of hemorrhagic shock, brain injury, and other causes of death were combined into a single control group for analysis. NDUFS8 alone demonstrated an area under the curve (AUC) of 0.828 (95% CI, 0.672–0.984; *p* = 0.0032), and SUCLG1 alone produced an AUC of 0.823 (95% CI, 0.674–0.972; *p* = 0.0037) (Figures 6E, F). The combined analysis of NDUFS8 and SUCLG1 achieved an AUC of 0.923 (95% CI, 0.830–1.000; *p* < 0.0001) (Figure 6G). Detailed ROC results are provided in Supplementary Data 2. Collectively, these findings suggest that NDUFS8 and SUCLG1, particularly when used in combination, may have exploratory discriminatory value as candidate ancillary markers for mechanical asphyxia. Given the limited sample size, these findings warrant confirmation in larger independent cohorts.

## Discussion

Mechanical asphyxia continues to pose significant challenges in forensic pathology regarding the determination of cause of death. The main difficulty lies in the lack of specific diagnostic markers in conventional gross and microscopic examinations. In a systematic review of hanging, Crudele et al. noted that the classic signs of asphyxia have limited diagnostic value (32); this limitation becomes more pronounced in complex cases, making the accurate identification of the cause of death particularly difficult. Previous studies have explored asphyxia-related biomarkers from multiple perspectives, including miRNAs, mRNAs, proteins, spectroscopy, and metabolomics (1, 14). At the level of myocardial mitochondria, a review by Hu et al. systematically summarized hypoxia-induced mitochondrial damage and its forensic significance, suggesting that mitochondrial dysfunction is a core molecular event shared by hypoxic and asphyxial deaths (33). Recent studies have utilized animal models combined with proteomic analysis to investigate potential biomarkers of mechanical asphyxia (13). Zheng et al. found changes in several mitochondrial complex I subunits (NADH dehydrogenase) and coagulation-related proteins in the myocardium of smothered mice (34), whereas Huang et al. identified ALKBH5, NAA10, and CLPB in rat and human myocardium. These differences among the reported proteins may arise from differences in species, control-group design, and analytical methods (13); nevertheless, most of the results converge on a common pathway of hypoxic stress and mitochondrial dysfunction. Notably, the changes in complex I subunits reported by Zheng et al. are consistent with the downregulation of NDUFS8—another complex I subunit—observed in this study, further supporting the view that impaired mitochondrial bioenergetic metabolism is a core feature of myocardial injury in mechanical asphyxia. To our knowledge, DIA proteomic data on human myocardium are still relatively scarce. In this study, a DIA-based proteomic analysis of human myocardial specimens was performed and combined with bioinformatic analysis and machine-learning methods. This strategy prioritized two mitochondria-associated proteins, NDUFS8 and SUCLG1, both of which were closely related to oxidative stress, and they were then assessed in human cardiac samples and further validated in animal models by western blot and immunohistochemistry (35).

The myocardium is one of the most oxygen-dependent organs (36). Accordingly, under acute and severe hypoxic conditions, the electron transport chain is particularly vulnerable to dysfunction, leading to reduced ATP production, redox imbalance, and mitochondrial impairment (37–41). In this study, proteomic analysis of myocardial tissue from mechanical asphyxia cases showed that KEGG enrichment was mainly related to the tricarboxylic acid cycle and oxidative phosphorylation, whereas GO biological process analysis was primarily associated with cellular respiration and energy metabolism. These findings suggest that myocardial mitochondrial bioenergetic metabolism is markedly disturbed during MA and may represent an important molecular pathological event. Previous studies have shown that mitochondrial dysfunction is a central mechanism in the initiation and progression of myocardial injury under ischemic and hypoxic conditions (42). Representative changes include succinate accumulation (43, 44), Ca^2+^ overload (45, 46), opening of the mitochondrial permeability transition pore (PTP) (47–50), and reverse electron transport (RET) at complex I, which promotes excessive reactive oxygen species production (51). These processes are often accompanied by altered mitochondrial dynamics, characterized by increased fission and reduced fusion (52–54). In addition, impaired mitophagy has been recognized as an important contributor to cardiovascular pathology, because failure to remove damaged mitochondria in a timely manner can further enhance oxidative stress and aggravate myocardial injury (55–57). Recent studies have provided additional mechanistic support for these observations. Hypoxia has been shown to reprogram mitochondrial metabolism through the METTL3–m6A–SLC25A11 axis and to promote ferroptosis in cardiomyocytes, linking hypoxia to mitochondrial transporter dysfunction and oxidative injury (58). Acute myocardial ischemia has also been reported to induce ferroptosis-related injury through the ALDH2/eIF3E signaling axis, indicating that redox imbalance and mitochondrial metabolic dysfunction are important determinants of cardiomyocyte fate (59). Taken together, the proteomic features identified in this study are consistent with current evidence on ischemic-hypoxic myocardial injury and support the view that mitochondrial metabolic dysfunction is a key feature of myocardial injury induced by mechanical asphyxia.

NDUFS8 (NADH:ubiquinone oxidoreductase core subunit S8) is a core subunit of mitochondrial complex I encoded by nuclear DNA. This protein harbors two conserved [4Fe−4S]^2+^/^1+^ iron–sulfur cluster motifs, that are critically involved in electron transfer and cellular bioenergetics (60, 61). Complex I serves as a significant site for electron leakage and oxidative damage under hypoxic and ischemic conditions. Hypoxic myocardial injury is often accompanied by complex I dysfunction and disrupted reactive oxygen species (ROS) homeostasis, suggesting that NDUFS8 is involved in hypoxia-related mitochondrial remodeling and plays a key regulatory role in hypoxic damage (61, 62). Datta et al. demonstrated in a time-resolved hypoxic proteomic study that NDUFS8 was coordinately downregulated alongside multiple oxidative phosphorylation-associated proteins, highlighting its role in the metabolic shift from mitochondrial aerobic respiration to glycolysis under hypoxic stress (63). Furthermore, Yang et al. reported in an acute myocardial hypoxia model that NDUFS8 contributed to MFN2-mediated enhancement of complex I activity and excessive ROS generation, positioning it as a pivotal factor in mitochondrial oxidative stress injury induced by acute hypoxia (64). Collectively, these findings underscore the notion that NDUFS8 may serve as a crucial molecular node linking mitochondrial dysfunction to energy-metabolic remodeling during hypoxic stress.

SUCLG1, the α subunit of succinyl-CoA synthetase/succinate-CoA ligase, plays a crucial role in the TCA cycle and mitochondrial substrate-level phosphorylation. In addition to its established metabolic function, SUCLG1 contributes to mitochondrial transcription and biogenesis by preventing the accumulation of succinyl-CoA and limiting the succinylation of mitochondrial RNA polymerase (POLRMT) (65). Moreover, Takada et al. reported that reduced succinyl-CoA levels in cardiomyocytes derived from chronic heart failure models directly compromise oxidative phosphorylation, highlighting the critical role of the succinyl-CoA/SCS axis in preserving mitochondrial energy homeostasis (66). In the context of acute hypoxia in myocardial tissue, the downregulation of SUCLG1 may indicate not only diminished TCA-cycle activity but also dysfunction at a critical metabolic node essential for mitochondrial energy production. Additionally, SUCLG1-associated features were enriched in pathways related to hypoxia, unfolded protein response, and the TNF-α signaling via NF-κB, suggesting that SUCLG1 downregulation is part of a broader adaptive stress network involving oxidative damage, proteostasis imbalance, and injury-response signaling.

This study also has several limitations. First, proteomic screening was based on a limited number of human samples and therefore carries a relatively high risk of false-positive findings. Owing to the small, high-dimensional discovery dataset, no candidate remained significant after FDR correction, and nominal *P* values were used only for exploratory screening. Nonetheless, both proteins showed large effect sizes, and the 95% confidence intervals for their log2 fold changes excluded zero. For NDUFS8, Cohen's *d* was −2.21, with a log2 fold-change 95% CI of −1.22 to −0.15, vs. hemorrhagic shock, and −3.14, with a 95% CI of −0.99 to −0.29, vs. brain injury. For SUCLG1, Cohen's *d* was −1.99, with a log2 fold-change 95% CI of −0.84 to −0.06, and −2.09, with a 95% CI of −0.76 to −0.07, respectively. The machine-learning analysis is also susceptible to overfitting and instability because it was performed in a discovery cohort of only 12 subjects, including four mechanical asphyxia cases. Furthermore, although NDUFS8 was identified as the leading candidate by the initial intersection, SUCLG1 was additionally retained after the MCC cutoff was extended to the top four. We acknowledge that this cutoff was selected *post hoc* and may have introduced selection bias. The resulting two-protein feature set should therefore be regarded as exploratory and requires confirmation in independent datasets. For these reasons, the proteomic and machine-learning analyses were used only for hypothesis generation and candidate prioritization, and the ROC analyses represent preliminary discriminatory performance rather than established diagnostic accuracy. Although the candidate proteins were further supported by protein–protein interaction analysis and subsequent experimental assessment, their discriminatory value and specificity remain to be established in larger, independent, and ideally multicentre cohorts. Second, although mechanical asphyxia, carbon-monoxide poisoning, and drowning all ultimately lead to hypoxia, they differ fundamentally in their underlying mechanisms. Mechanical asphyxia is characterized by violent struggling and a sharp increase in oxygen consumption and is caused directly by external mechanical factors, such as airway obstruction, neck compression, or thoraco-abdominal compression. By contrast, drowning results from liquid obstruction of the airways and alveoli, whereas carbon-monoxide poisoning involves carboxyhaemoglobin formation and direct interference with cellular respiration. Moreover, drowning and carbon-monoxide poisoning can generally be supported by positive autopsy or toxicological findings, whereas the diagnosis of mechanical asphyxia is often exclusion-based because of the lack of a specific diagnostic finding. These mechanistic and diagnostic differences provide a biological basis for the comparatively characteristic downregulation of NDUFS8 and SUCLG1 in mechanical asphyxia. Nevertheless, reduced expression of these proteins was reproducibly validated only in the animal models, in which the cause of death and postmortem interval could be controlled. Although the same direction of change was observed in the human samples by Western blotting and immunohistochemistry, the limited cohort size means that these findings remain preliminary. NDUFS8 and SUCLG1 should therefore be regarded only as preliminary potential markers of mechanical asphyxia, and their discriminatory value requires confirmation in larger, independent, and ideally multicentre cohorts. Third, mechanical asphyxia comprises several distinct death mechanisms, and this study covered only manual strangulation, ligature strangulation, hanging, and smothering; not all subtypes were included, and the number of cases in each subtype was limited. More importantly, real cases often involve a combination of death mechanisms, and atypical or mixed-mechanism situations, such as autoerotic death, still need further validation in larger cohorts in the future (67). Fourth, postmortem biochemical indicators are inevitably affected by the postmortem interval, environmental temperature, underlying diseases, and inter-individual variability, and metadata such as the agonal period, resuscitation attempts, and comorbidities were missing for some cases. In real forensic practice, most bodies are already deceased when discovered, and the agonal duration and any resuscitation attempts are frequently not documented in autopsy records. Because of these missing data, we were unable to incorporate all of these variables into the analysis under ideal conditions, and the potential confounding effect of such factors on marker expression could not be fully evaluated. Future studies should enlarge the sample size and prospectively collect more complete metadata, including the agonal period, resuscitation attempts, and relevant comorbidities, so that these factors can be systematically accounted for. Fifth, female victims account for a relatively high proportion of mechanical asphyxia cases, whereas the animal model in this study used only male mice, which may limit the generalizability of the findings.

In summary, we combined high-throughput proteomics with machine learning to identify candidate molecular markers of mechanical asphyxia, with a focus on mitochondrial metabolic dysfunction and oxidative stress. NDUFS8 and SUCLG1 emerged as the leading candidates and offer mechanistic interpretability. They may serve as candidate ancillary markers of mechanical asphyxia. These findings provide preliminary evidence to support further investigation of molecular markers for mechanical asphyxia.

## Data Availability

The datasets presented in this study can be found in online repositories. The names of the repository/repositories and accession number(s) can be found below: https://www.iprox.cn//page/project.html?id=IPX0018430000.
