# Supplementary material for: Combined machine learning identification and experimental validation of NDUFS8 and SUCLG1 as potential biomarkers for mechanical asphyxia
Source: Front Med (Lausanne). 2026 Aug 26;13:1861399. doi: 10.3389/fmed.2026.1861399 (PMC13553469; doi:10.3389/fmed.2026.1861399)
Supplement: Supplementary file 1 [file Supplementary_file_1.pdf]

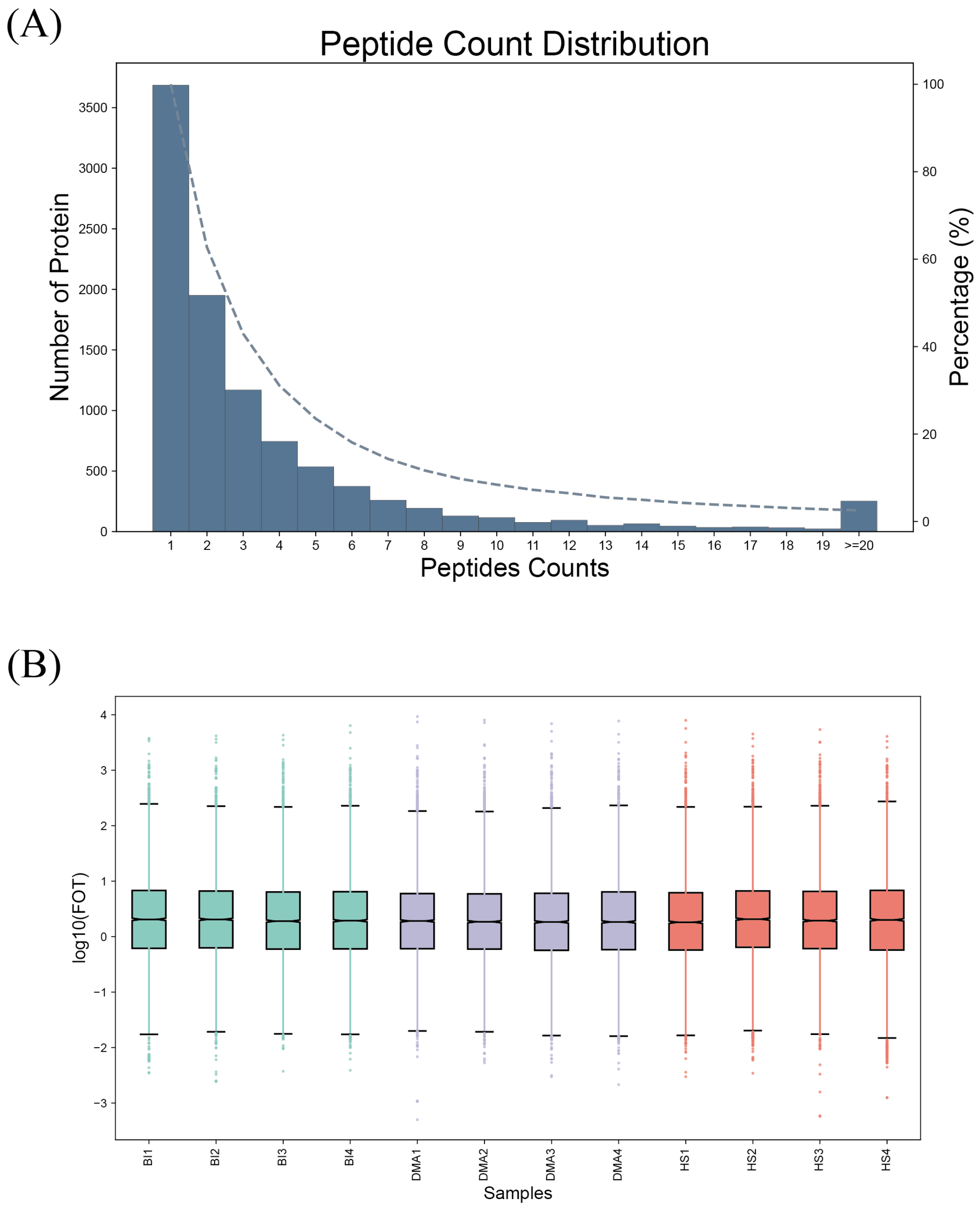

Supplementary Figure 1. Quality assessment of the DIA proteomic dataset. (A) Distribution of peptide counts per identified protein. Bars show the number of proteins identified with a given number of peptides, and the dashed line shows the cumulative percentage. (B) Distribution of protein abundance across the 12 discovery samples. Notched box plots show the log10-transformed FOT values for the brain injury (BI1–BI4), mechanical asphyxia (MA1–MA4), and hemorrhagic shock (HS1–HS4) groups. In each box the central line denotes the median, the box the interquartile range, and the whiskers the 1.5× interquartile range.

(A)

(B)

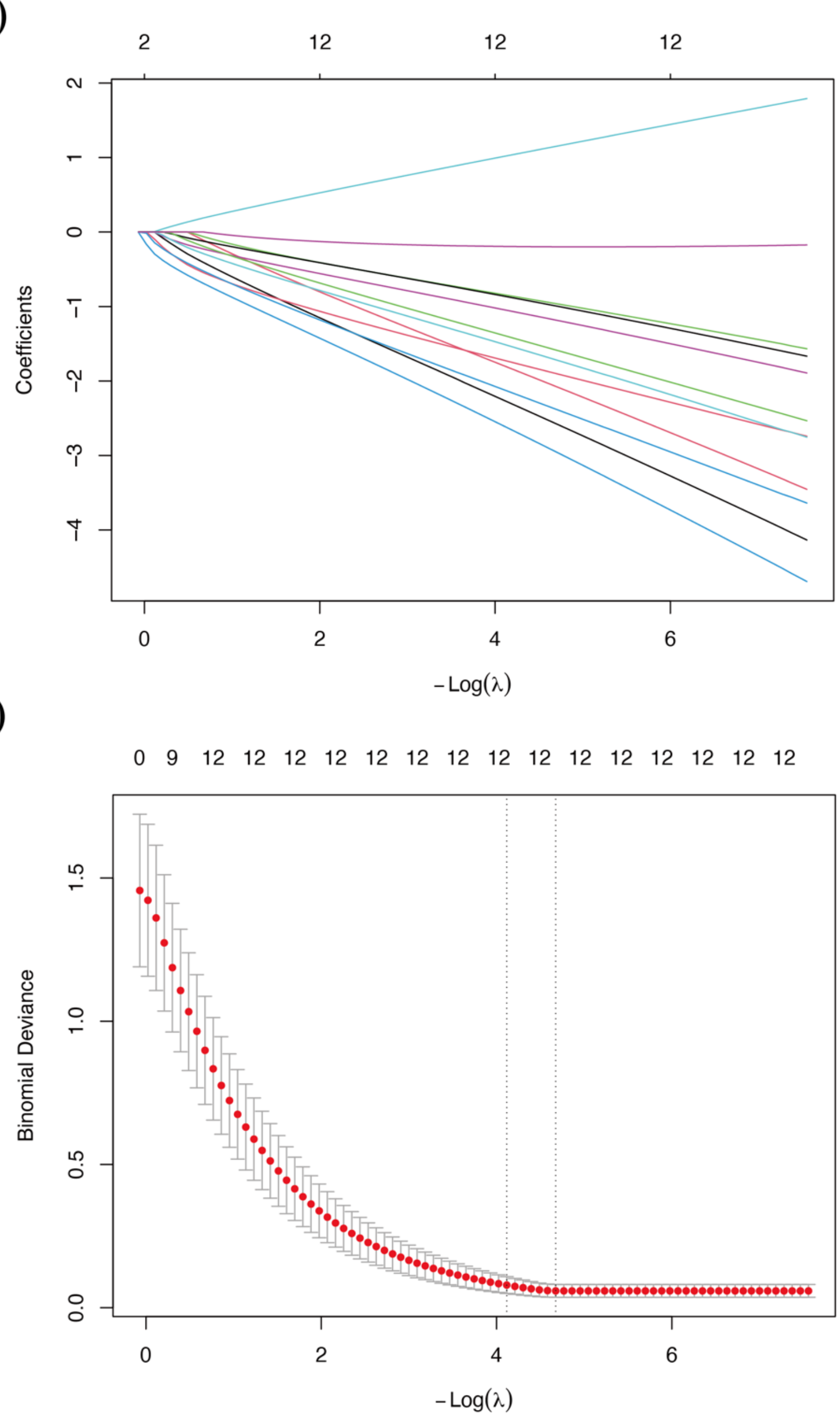

Supplementary Figure 2. Elastic-net regression for candidate-protein prioritization. (A) Coefficient profiles of the candidate proteins plotted against −log(λ). (B) Selection of the optimal penalty parameter (λ) by five-fold cross-validation, with binomial deviance plotted against −log(λ). The mixing parameter α was tuned from 0 to 1 and set to the optimal value of 0.4. Analyses were performed on the log2-transformed abundances of the 12 mitochondria- and oxidative stress–related proteins across the 12 samples.

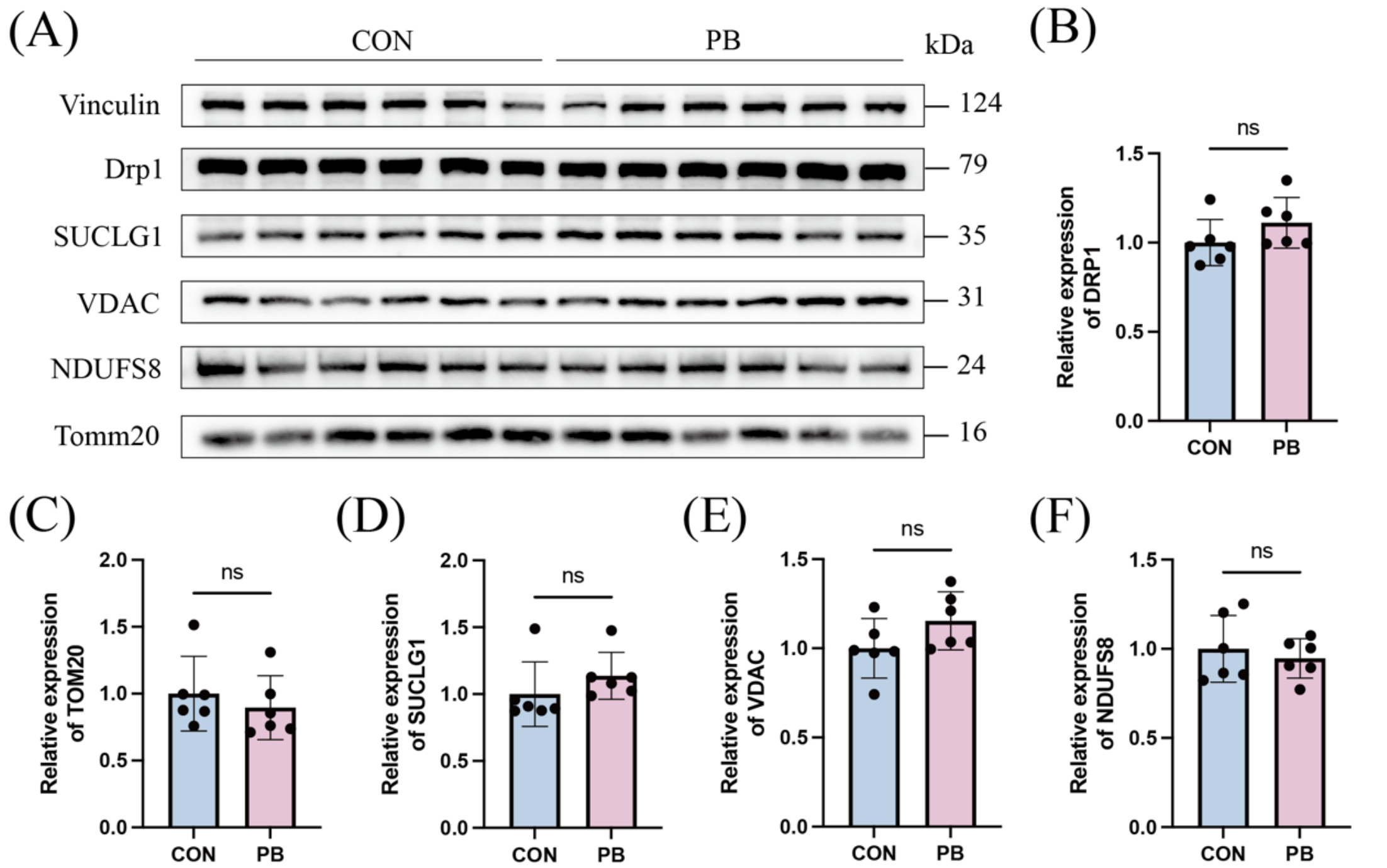

Supplementary Figure 3. Sodium pentobarbital anaesthesia does not significantly alter the expression of mitochondria-related proteins in the mouse myocardium. (A) Representative Western blots of Drp1, SUCLG1, VDAC, NDUFS8, and Tomm20 in myocardial tissue from the control (CON) and sodium pentobarbital (PB) groups. Vinculin was used as the loading control. (B–F) Quantification of the relative expression of Drp1 (B), Tomm20 (C), SUCLG1 (D), VDAC (E), and NDUFS8 (F). Data are presented as mean ± SD (n = 6 mice per group). Differences between groups were assessed by Student's t-test. ns, not significant.

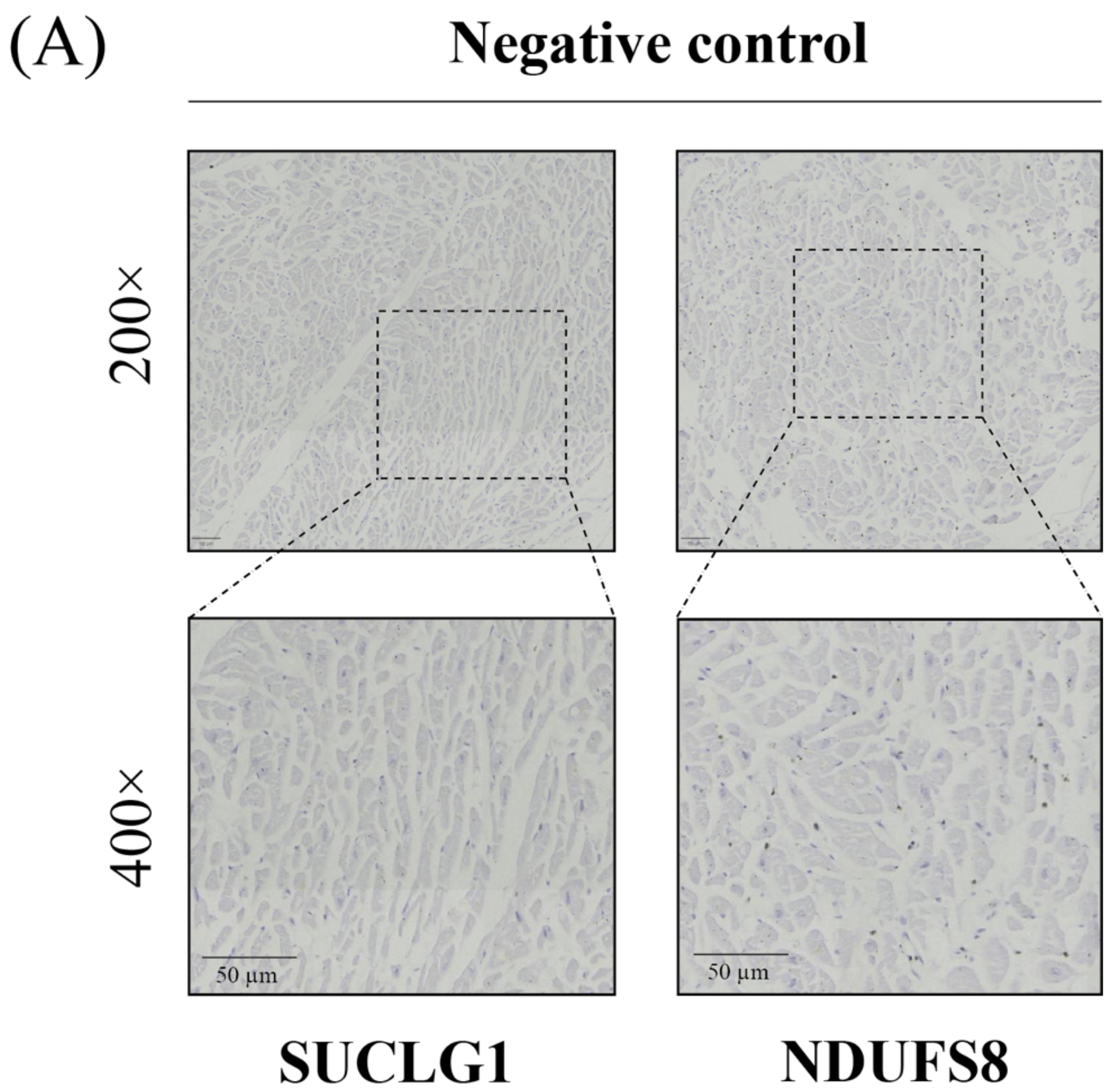

Supplementary Figure 4. Negative controls for immunohistochemical staining of SUCLG1 and NDUFS8 in human myocardium. (A) Representative images of negative-control sections for SUCLG1 (left) and NDUFS8 (right), obtained from a control-group human sample, in which the primary antibody was replaced with PBS. No specific immunostaining was observed. The upper panels were acquired at 200× magnification, and the lower panels show the boxed regions at 400× magnification. Scale bars, 50 μm.
