# Supplementary material for: Combined machine learning identification and experimental validation of NDUFS8 and SUCLG1 as potential biomarkers for mechanical asphyxia
Source: Front Med (Lausanne). 2026 Aug 26;13:1861399. doi: 10.3389/fmed.2026.1861399 (PMC13553469; doi:10.3389/fmed.2026.1861399)
Supplement: Supplementary file 2 [file Data_Sheet_2.docx]

**Supplementary Data 2. ROC analysis of SUCLG1 and NDUFS8.**

| **Parameters** | **Cut-off** | **Sensitivity** | **Specificity** | **Youden Index** | **LR+** | **LR−** | **AUC** | **Std. Error** | **95% CI** | **P-value** |
| --- | --- | --- | --- | --- | --- | --- | --- | --- | --- | --- |
| SUCLG1 | 0.1818 | 0.8182 | 0.6842 | 0.5024 | 2.59 | 0.27 | 0.8230 | 0.07616 | 0.6737–0.9722 | 0.0037 |
| NDUFS8 | 0.1649 | 0.7273 | 0.8421 | 0.5694 | 4.61 | 0.32 | 0.8278 | 0.07961 | 0.6717–0.9838 | 0.0032 |
| SUCLG1 & NDUFS8 | 0.4451 | 0.8182 | 0.8947 | 0.7129 | 7.77 | 0.20 | 0.9234 | 0.04751 | 0.8303–1.000 | 0.0001 |

ROC analysis was performed on 30 immunohistochemistry samples (11 mechanical asphyxia and 19 controls). LR+, positive likelihood ratio; LR−, negative likelihood ratio; AUC, area under the curve; CI, confidence interval.
